# A membrane-free spot-plating protocol for *Agrobacterium*-mediated transformation of diverse yeasts

**DOI:** 10.1371/journal.pone.0359072

**Published:** 2026-10-08

**Authors:** Matthew R. Incha, Matthew J. Szarzanowicz, Gina M. Geiselman, Amy Lanctot, Paul A. Adamczyk, Valentina E. Garcia, Robin A. Herbert, Mitchell G. Thompson, Patrick M. Shih, John M. Gladden, Di Liu

**Affiliations:** 1 Agile BioFoundry, Emeryville, California, United States of America; 2 Sandia National Laboratories, Livermore, California, United States of America; 3 Joint BioEnergy Institute, Emeryville, California, United States of America; 4 Environmental Genomics and Systems Biology Division, Lawrence Berkeley National Laboratory, Berkeley, California, United States of America; 5 Department of Plant and Microbial Biology, University of California, Berkeley, California, United States of America; 6 Innovative Genomics Institute, Berkeley, California, United States of America; Cornell University, UNITED STATES OF AMERICA

## Abstract

*Agrobacterium*-mediated transformation (AMT) is a critical method for genetic manipulation of non-model fungi, yet it remains a laborious and inefficient technique. When co-cultured in acetosyringone-supplemented induction medium, *Agrobacterium* transfers DNA directly into yeast cells using its virulence machinery. Membrane filters are commonly used to support the co-culture of yeast and *Agrobacterium* on agar plates, however some reports demonstrate that these filters are unnecessary for specific yeast species. Here we confirm across diverse budding yeasts that membrane filters are not necessary for effective AMT. Concentrating the cells via centrifugation and “spotting” the cell pellet directly onto the induction medium proved effective. This reduces hands-on time to 15 minutes and eliminates filter cost. In the oleaginous basidiomycete yeast, *Rhodotorula toruloides*, this simplified method increases transformation efficiency by 66% to 2,500 transformants per 10^6^ recipient cells. We further optimized the *Agrobacterium*:*Rhodotorula* cell ratio and culture resuspension volume to achieve more than 200,000 CFU per transformation representing a 2–3 fold improvement over previously implemented protocols. This spot-plating method was successfully applied to seven yeast species of a distantly related phylum, Ascomycota, including one for which genetic transformation has not previously been reported, *Botryozyma nematodophila.* This approach highlights the broad applicability of the spot-plating method across diverse yeast systems. Furthermore, this method could facilitate high-throughput transformation workflows that are critical for genome-scale functional studies.

## Introduction

Fungal transformation is critical for basic biological research and applied technical applications in pharmaceuticals, bioproduction, and food fermentation. The investigation and biotechnological use of these important organisms is often hindered by our inability to efficiently transform heterologous genetic material into these species. Unlike *Saccharomyces* spp. and *Komagataela* (*Pichia*) spp., many fungal species are recalcitrant to transformation via traditional methods of electroporation or chemical transformation, often requiring laborious protoplasting protocols [1]. This makes *Agrobacterium-*mediated transformation (AMT) an advantageous method for introducing heterologous DNA to these species and making genetic knockouts.

AMT leverages the naturally pathogenic relationship between *A. tumefaciens* and plants. In a mechanism analogous to bacterial conjugation, *Agrobacterium* transmits DNA encoding oncogenes into plant cells inducing tumor formation, the characteristic symptom of crown-gall disease. The oncogenes and the virulence genes (*vir* genes) that enable their transmission are encoded on the tumor-inducing (Ti) plasmid of *Agrobacterium* [2], [3]. The oncogenes are located in a region called the transfer-DNA (T-DNA) and are flanked by defined DNA sequences known as the right border (RB) and left border (LB). Facilitated by the virulence proteins, the T-DNA sequence between these borders is excised, transferred into recipient cells, escorted to the nucleus, and integrated into the host genome [4].

Capitalizing on *Agrobacterium*’s plant-genome transformation capacity, plant biologists domesticated *Agrobacterium* by removing oncogenes from the Ti plasmids and porting the LB and RB sequences into smaller synthetic plasmids known as binary vectors [5–7]. By cloning transgenes between these conserved border sequences, biologists are able to stably introduce novel transgenic sequences directly into the plant genome, revolutionizing plant genetics and engineering [8]. Later, AMT was implemented to transgenically modify fungal species [9]. In this pivotal work of Bundock et al., *Saccharomyces cerevisiae* and *Agrobacterium* were mixed and added directly onto filters placed on an induction medium plate [9], a protocol presumably modified from standard practice for bacterial conjugation [10]. However, since this first description, many groups have employed vacuum filtration of the co-culture to embed larger quantities of cells onto the membrane [11].

While filter membranes are helpful for handling filamentous fungi after co-cultivation [12], the use of filters for budding-yeasts appears, at first glance, unnecessary. The additional step of vacuum filtration adds time, labor, batch-variability, and risk of contamination. Mitigating these challenges is important for the widespread adoption of this transformation method. Modifications to the protocol have been made to improve transformation efficiency, such as changing the choice of filter polymer and the method of embedding cells onto the filter [13]. Despite this, only a few reported methods in *Cryptococcus* and *Rhodotorula* genetics studies bypass the filter altogether and spot the mixture of *Agrobacterium* and yeast directly onto the induction medium [14, 15]. Here, we demonstrate that a spot-plating method omitting filter membranes and vacuum filtration can be optimized and broadly deployed across diverse yeast species of distantly related basidiomycetes and ascomycetes.

## Results and discussion

### Spot plating improves transformation efficiency, reduces protocol time, and does not impact transformation outcomes compared to filtered transformations

In this study, we primarily focused on the basidiomycete yeast *Rhodotorula toruloides* IFO0880 (NBRC 0880/NRRL 1588), as we routinely achieve more than 50,000 colonies per transformation in this species using AMT with vacuum filtration. All transformations in this host were conducted with the binary vector plasmid, pVL_sR2rn_f14rg, containing the *R. graminis* WP1 TUB2 promoter driving a nourseothricin resistance gene, *nrsR*, and the P14 promoter, driving a codon optimized sfGFP (rg) [16].

The standard protocol is briefly described here: *Agrobacterium tumefaciens* strain EHA105 carrying a desired binary vector is cultured in LB to an OD of ~1. The culture is then centrifuged, washed, and incubated overnight in an equal volume of induction medium. The target yeast is separately cultured to an OD of ~1 in YPD (4.5E7 cells for *Rhodotorula*). One milliliter of yeast is pelletted, the YPD is aspirated and the pellet resuspended with one milliliter of acetosyringone-induced *Agrobacterium*. The *Agrobacterium*-yeast mixture is then pulled onto a 0.45 µm mixed cellulose ester filter under vacuum, and the filter is placed onto an induction medium plate for 4–5 days. After growth on the induction medium, the co-culture is then removed from the filter, resuspended in YPD, and culture dilutions are plated onto selective medium plates with the addition of beta-lactams (cefotaxime, carbenicillin, or timentin) to prevent *Agrobacterium* growth.

The process of vacuum-pulling and drying the mixed culture of yeast and *Agrobacterium* onto a filter membrane is incredibly laborious and time-consuming. With standard bench-top vacuum pumps (0.1–0.4 bar), this step can vary from 10 minutes to an hour for each filter, hindering higher-throughput transformation workflows. Using a single filtration apparatus, making a pooled library of 2 × 10^6^ transformants would require dozens of filters and hours of hands-on labor [17]. To demonstrate this labor requirement, three researchers conducted and timed AMT of *Rhodotorula* using two distinct protocols using the same batches of *Agrobacterium* and *Rhodotorula*: the vacuum filtration protocol and a modified protocol wherein the cell mixture was added directly onto the induction medium. Two of the three researchers completed 6 replicates of the protocol including vacuum filtration in approximately three hours. One researcher had a fault in their vacuum apparatus that could not be identified, and they completed 6 filtration replicates in 5 hours. This extended period resulted in extensive contamination, and their data were unviable.

In contrast, the modified protocol that omits membrane filters and vacuum filtration took approximately 15 minutes to complete 6 replicates for all three researchers. This method simply requires centrifuging the mixture of *Rhodotorula* and *Agrobacterium*, aspirating the supernatant, resuspending the cell pellet, and pipetting the entire mixture as a droplet directly onto the induction medium plate (“spot-plating”). These plates were then left to dry for 10 minutes in a biosafety cabinet. After growth on the induction medium for 4–5 days, this protocol yields a spot of mixed cells approximately 1–2 cm in diameter.

Notably, the spot-plating method resulted in significantly more transformants than the vacuum filtration method. The vacuum filtration method yielded an average of 67,233 ± 14,269 CFU per transformation (range: 50,400–95,200) and an efficiency of 1,488 transformants per 10^6^ of starting *Rhodotorula* cells. The spot method produced 111,600 ± 19,782 CFU (range: 64,000–143,040) and an efficiency of 2,480 transformants per 10^6^ of starting *Rhodotorula* cells (Fig 1A). This represents a 66% increase in transformation efficiency for the spot-plating method relative to the standard vacuum filtration protocol (*p* < 0.001, Welch’s two-tailed *t*-*t*est). This improvement was consistent across the transformation efficiencies obtained by both researchers, indicating the method’s reproducibility.

**Fig 1:**
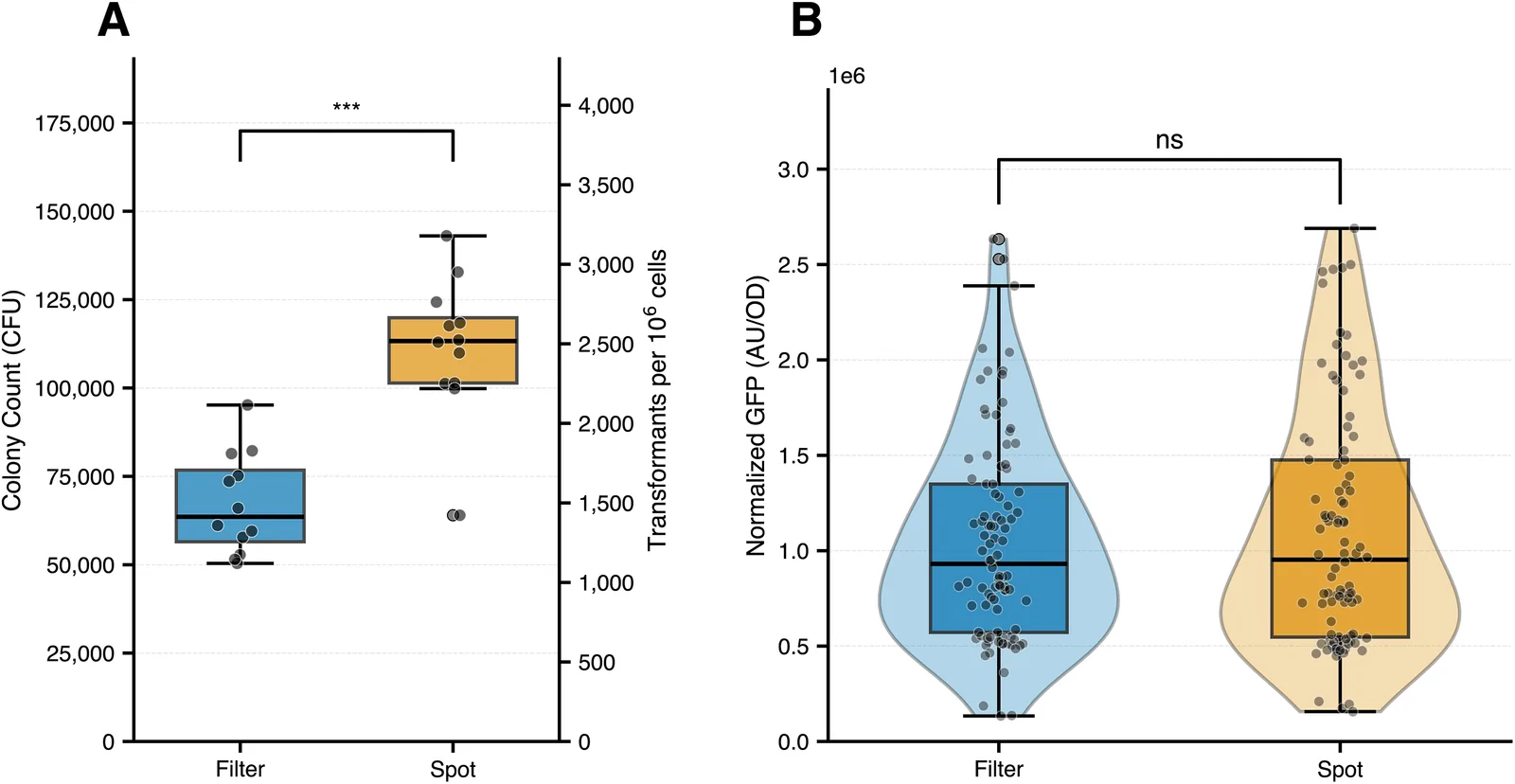
Comparison of filter and spot methods in *Rhodotorula toruloides.* **A.** Transformation results in terms of number of CFUs from the filter and spot plating methods (n = 12 filters and 12 spots, p = 0.000004 Welch’s t-test). **B.** Comparison of the transformation outcomes in terms of GFP intensity from randomly selected colonies from transformants in 1A (n = 96 per group, p = 0.5364 Welch’s t-test, p = 0.99 Mann-Whitney U). All transformations were conducted with the binary vector pVL_sR2rn_f14rg.*** p < 0.001 Welch’s t-test.

Transformation efficiency is not the only metric critical for a transformation protocol, as the genetic outcome of the transformation is also an essential criterion. To test if the transformation outcome was equivalent between the two protocols, we estimated the transgene copy number in 96 colonies from each method using fluorescence intensity as a proxy for GFP insertion events [18]. Transformed colonies were cultured in YPD, and their fluorescence was measured using a plate-reader. The levels of fluorescence in these populations were indistinguishable, suggesting that both methods yielded similar copy numbers of transgenic integration events (Fig 1B). Single-cell GFP fluorescence was confirmed using fluorescence microscopy (S1 Fig).

### *Agrobacterium*:Yeast ratio contributes to transformation efficiency

To further optimize the spot-plating method, we systematically tested how cell densities of *Rhodotorula* and *Agrobacterium* affect transformation efficiency. Cell quantities are reported as OD·mL (OD_600_ × volume in mL), where 1 OD·mL represents the cell content of 1 mL of culture at OD_600_ = 1. Cultures were retrieved at OD_600_ = 1 before use, and varying volumes were concentrated or diluted to achieve the indicated OD·mL values. Three cell densities (0.2, 1, and 5 OD·mL) for both species were tested across nine pairwise conditions. All 9 combinations produced viable transformants, but efficiency varied dramatically (34–1,594 CFU per 10^6^ *Rhodotorula* cells). The highest yield of transformants was achieved with the highest combined cell density (5 OD·mL each; mean: 218,333), representing a 3-fold improvement over the standard density (1 OD·mL each; mean: 71,750 CFU) (Fig 1A). Transformation efficiency was slightly lower at the highest cell density ratio compared to the standard density, though this difference was not statistically significant (Fig 2B). When the density of *Rhodotorula* cells was high (5 OD·mL), efficient transformation required proportionally higher densities of *Agrobacterium* (5 OD·mL) (Fig 2A,B). This may be attributed to reduced *Agrobacterium*-*Rhodotorula* cell-to-cell contacts due to occlusion by the high quantity of *Rhodotorula* cells.

**Fig 2:**
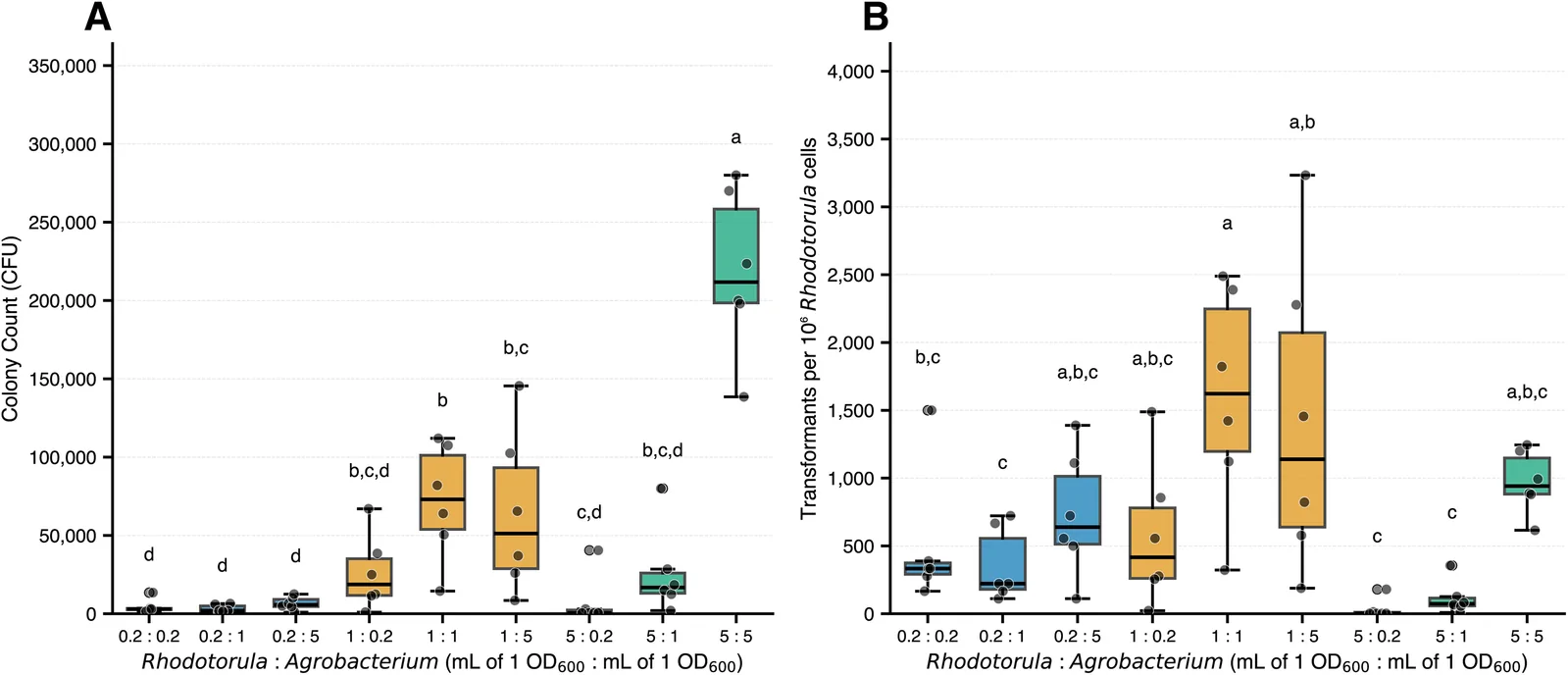
Cell ratio and spot volume effects on transformation efficiency. **A.** Total colonies per transformation with different proportions of *Agrobacterium* and *Rhodotorula* in terms of OD·mL. Letters above each group denote statistically distinct groups based on Tukey's HSD post-hoc test (familywise error rate (FWER) = 0.05) following a significant one-way ANOVA (F = 30.56, p < 0.0001). Groups sharing a letter are not significantly different from one another. **B.** Transformation efficiency in terms of transformants per 10^6^ *Rhodotorula* cells with different proportions of *Agrobacterium* and *Rhodotorula*. Letters above each group denote statistically distinct groups based on Tukey's HSD post-hoc test (FWER = 0.05) following a significant one-way ANOVA (F = 5.55, p < 0.0001). Groups sharing a letter are not significantly different from one another.

The size of the co-culture spot could additionally influence cell-to-cell contact. To evaluate this, we tested three resuspension volumes (20 μL, 75 μL, and 150 μL) using the standard OD·mL cell densities (1 OD·mL each). The 75 μL volume produced the highest colony counts (mean: 92,600 CFU), compared to 87,933 for 20 μL and 63,733 for 150 μL; however, these results were not statistically significant (S2 Fig). The resulting spots are of increasing diameter as the volume increases. Upon drying, cell-cell contact remains sufficient for effective AMT across the range of volumes tested.

### Unfiltered spot-plating method is effective across species in diverse genera of a distant phylum

Having validated the spot plating method in the basidiomycete *Rhodotorula toruloides*, testing it on members of the distant phylum, Ascomycota, would demonstrate its broader potential. To this end, we first compared the filter and spot-plating methods using *Saccharomyces cerevisiae* and *Kluyveromyces marxianus*. *S. cerevisiae* was the first fungus transformed via AMT, while *K. lactis*, a close relative of *K. marxianus,* was also among the earliest [9], [19]. For *S. cerevisiae*, we constructed a plasmid (pVR_sATk_fS3sg_HR) containing the *Ashbya gossypii* TEF1 promoter driving a G418 resistance cassette, the *S. cerevisiae* TDH3 promoter driving sg12 GFP expression, and homology arms targeting the *tau* locus of chromosome XVI. Transformation of *K. marxianus* was conducted with pVR_sATk_fS3sg, an otherwise identical plasmid without homology arms. *K. marxianus* strains have demonstrated preference for NHEJ mechanisms similar to many environmental yeast strains making this non-targeting plasmid an effective test-case [20, 21]. Contrary to the results in *Rhodotorula*, the spot-plating method yielded fewer colonies for both hosts. Though this decrease was not statistically significant for *S. cerevisiae* (p > 0.05) but was highly significant for *K. marxianus* (p < 0.001) (Fig 3). *S. cerevisiae* yielded 99,400 ± 39,972 and 66,400 ± 19604 transformants in the filter and spot-plating methods, respectively. *K. marxianus* yielded 3,860 ± 811 and 880 ± 421 transformants in the filter and spot-plating methods respectively. However, the 5 replicates for each host required between 15–45 minutes for each filter to be pulled and dried (3 hours total), while the spot-plating was conducted in parallel and finished in 30 minutes (accounting for dry-time in a biosafety cabinet). The reduced efficiency when spot-plating these hosts could be due to any number of differences relative to *Rhodotorula toruloides*, potentially highlighting a fundamental difference in the physiology of basidiomycetes and ascomycetes or in the secretion of metabolites.

**Fig 3:**
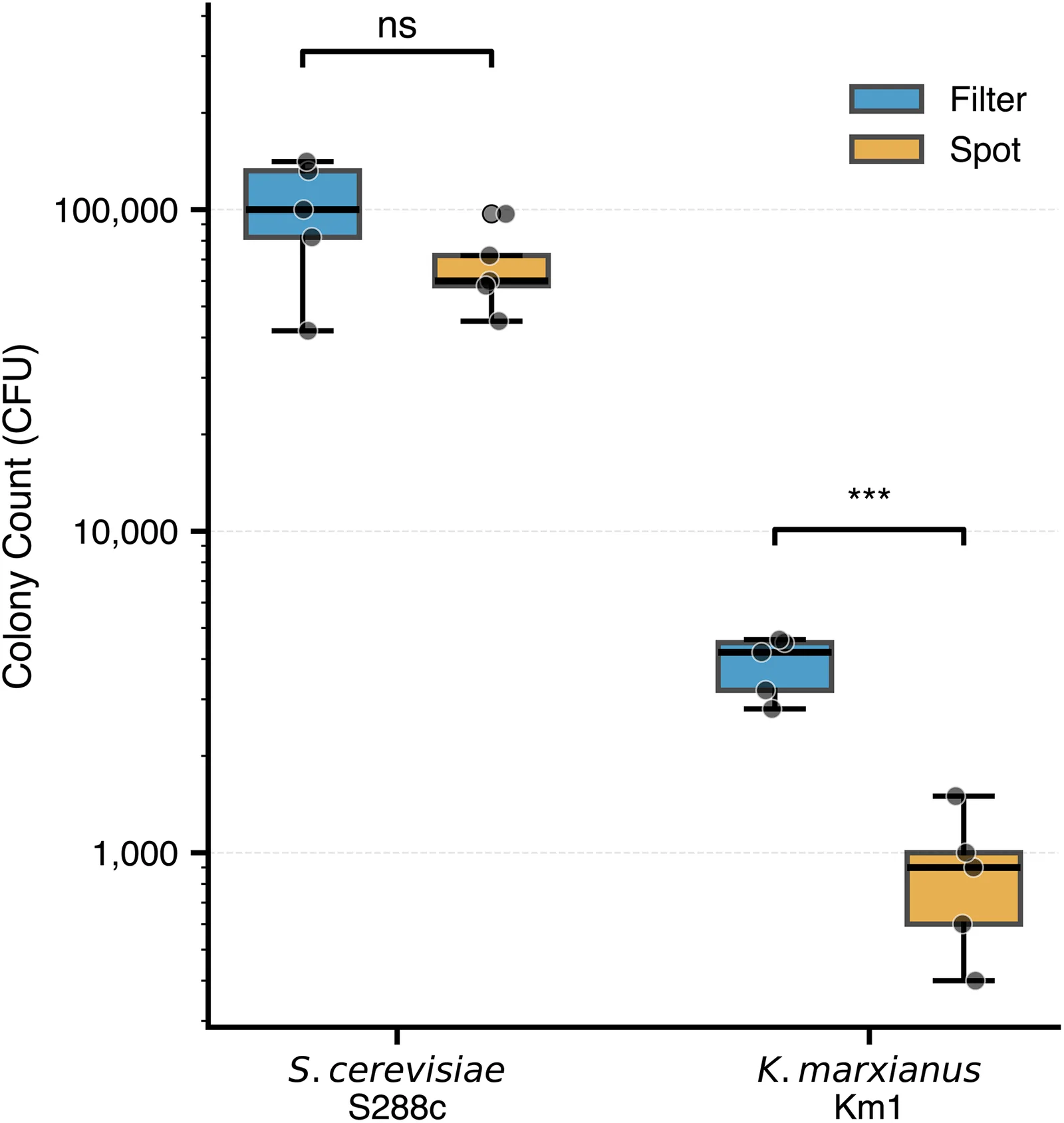
Comparison of filter and spot methods in *S. cerevisiae* and *K. marxianus.* Transformation results in terms of total number of CFUs from the filter and spot-plating methods. Results were from 1 mL of 1 OD culture for both *S. cerevisiae* and *K. marxianus*. *** p < 0.001 Welch’s t-test.

As a final demonstration for the unfiltered protocol, another five phylogenetically diverse ascomycete yeasts were transformed: *Metschnikowia pulcherrima*, *Komagataella phaffii* (*Pichia pastoris*), *Ogataea parapolymorpha*, *Lipomyces tetrasporus*, and *Botryozyma nematodophila*. To our knowledge, only *L. tetrasporus* has been transformed via AMT. Members of the other genera have been transformed with alternative methods (i.e., PEG-mediated protoplasting, lithium acetate chemical transformation, or electroporation) [22–25]. However, genetic transformation of *Botryozyma spp.* has not been previously reported (Fig 4A and 4B). Nourseothricin was found to be an effective antifungal against all species, so each was transformed with variations of the nourseothricin resistance (*nrsR*) gene with different promoters: the wildtype *Streptomyces nourseii nrsR* (n) two codon optimized *nrsR* variants (on and ln), the *A. gossypii* TEF1 promoter (AT), and the *L. starkeyii* TRPC (LR) promoter.

**Fig 4:**
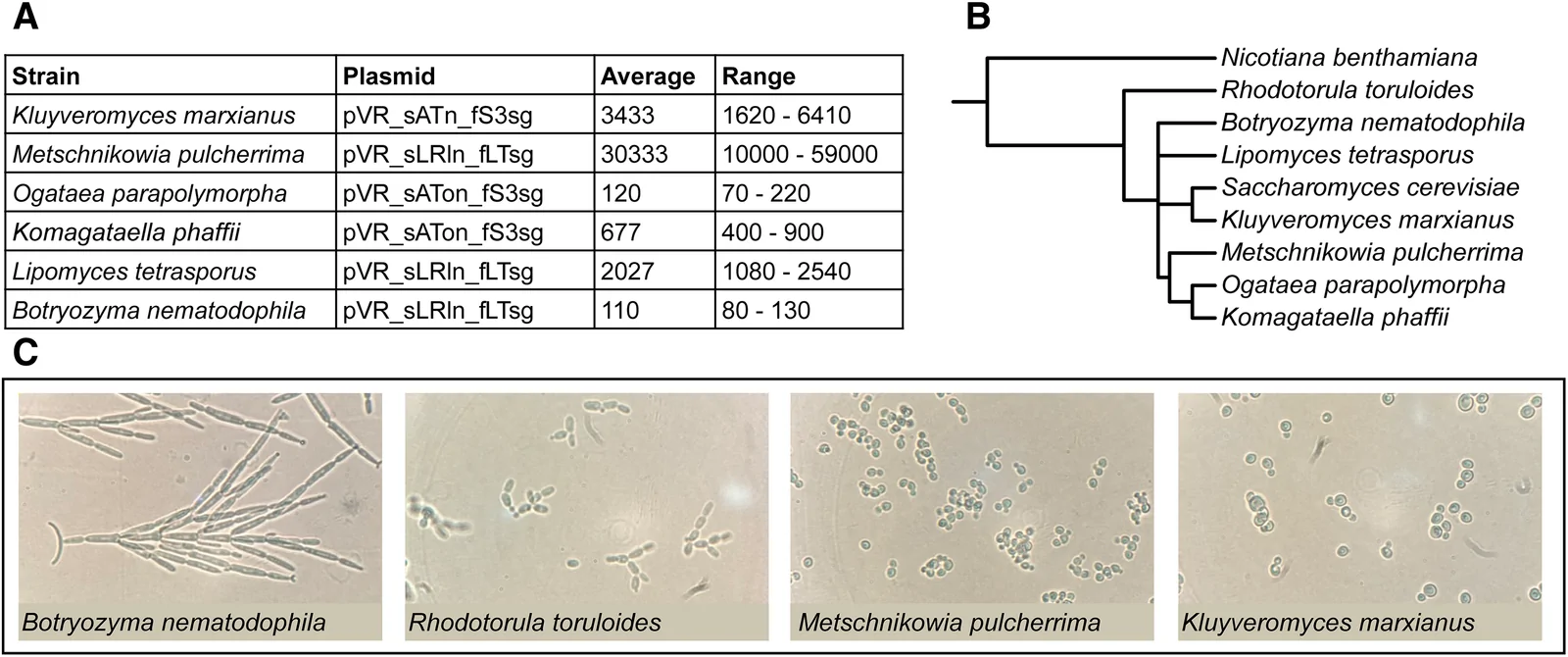
Transformation of diverse Ascomycete yeasts. **A.** Table of select transformation results for diverse Ascomycete yeasts. Complete table available in Table S2 in in S1 File. All transformations were conducted using the spot-plating method with *Agrobacterium*:yeast OD·mL ratios of 1:1. **B.** Family tree of all tested microbes with *Nicotiana benthamiana* as an outgroup to root the tree. The tree was constructed via phyloT v2 using NCBI taxonomy [26]. **C.** Microscopy illustrating the diverse morphologies of select tested microbes. Microscopy of all tested microbes is available in S4 Fig.

All variants of the selection cassette worked to varying degrees in all hosts except *M. pulcherrima* (Table S2 in S1 File). *M. pulcherrima* is a CTG-Ser yeast and could only be successfully transformed with the codon optimized *nrsR* cassettes, both of which lacked CTG codons [23]. Transformation yields varied by species (from <100 to >100,000 colonies), possibly reflecting differences in morphology (Fig 4C), cell wall composition, T-DNA integration efficiency, selection marker expression, or preferred DNA repair mechanisms (HR vs NHEJ). Nonetheless, the fact that all seven species could be transformed via this protocol demonstrates that the unfiltered method is broadly applicable across diverse yeast lineages.

Due to the novelty in transforming *M. pulcherrima* and *Botryozyma nematodophila*, 8 transformants from each vector were propagated on fresh plates and screened via colony PCR. In all cases, 8 out of 8 transformants yielded the desired PCR product (S3 Fig). While we simply quantified the total number of resulting transformants and not the efficiency, future work on host-specific optimization could evaluate this metric.

## Conclusions

The spot-plating method reported and optimized here streamlines AMT of non-model fungal species, a historically labor-intensive, time-consuming, and inconsistent process. By eliminating the vacuum and filter entirely, this protocol reduces hands-on time from several hours to approximately fifteen minutes for 12 transformations, notably increasing the speed and feasibility of AMT for large-scale functional genomic experiments. We furthermore found that this method improved transformation efficiency relative to prior filter plating protocols by up to 66% for *Rhodotorula toruloides*. By systematic optimization of cell densities and resuspension volumes, we obtained more than 200,000 total transformants per transformation. This enables transformation with sufficient coverage for the construction of large libraries.

Our successful transformation of seven yeast species spanning diverse genera from two phyla using spot-plating confirms that the unfiltered approach is not limited to well-characterized model organisms but functions as a potential platform for onboarding molecular biology tools into non-model hosts. Successful transformations in *Botryozyma nematodophila* and *Metschnikowia pulcherrima* are of particular interest for their unique morphology and under-explored biotechnological potential respectively [27–29].

Despite its lower or equivalent transformation efficiency relative to filter-plating in *K. marxianus* and *S. cerevisiae*, spot plating is the more practical method, requiring far less hands-on time (15 minutes vs. 2 or more hours for 6 replicates) and enabling parallel processing by eliminating the need for vacuum pump apparatuses and filters altogether. The reason for the discrepancy in efficiency relative to *Rhodotorula* may be due to any number of factors. Notably, the *Rhodotorula* cell wall contains distinct exopolysaccharides and an outer cell capsule, while *S. cerevisiae* and *K. marxianus* produce fermentation products (lactate, acetate, or ethanol) [30–33]. While we did not test the filter method, *Metschnikowia pulcherrima* produces pulcherrimin, an iron chelator with antimicrobial properties, which could benefit one method over the other [34]. Though these hypotheses have not been tested, they would be interesting lines of investigation in future work.

We did not conduct direct comparisons between spot plating and the standard method for all the yeasts explored here, therefore it is possible each host will perform differently using either method. However, due to its ease of application and the elimination of filters, the simplified protocol can be easily scaled to account for any deficits in efficiency. Additionally, optimization of the unfiltered method can be easily conducted by researchers investigating new hosts, following the framework described here.

This protocol brings large-scale functional genomics experiments in recalcitrant yeast species within practical reach for researchers who previously lacked the capacity to attempt them. The straightforward method of spotting a mixture of *Agrobacterium* and yeast onto an induction medium enables automated workflows, accelerating investigation and engineering in genetically recalcitrant yeasts.

## Methods

### Media, chemicals, and culture conditions

Bacteria were routinely propagated in Luria-Bertani (LB) medium (BD Biosciences, USA). Wildtype yeast were routinely propagated in yeast extract, peptone, dextrose (YPD) medium without additional supplementation, unless otherwise noted (Sigma-Aldrich, USA). *Escherichia coli* and *Agrobacterium tumefaciens* were grown at 37 °C and 30 °C, respectively. Liquid cultures were shaken at 200 RPM.

Antibiotics and concentrations used are as follows: kanamycin (50 mg/L, Teknova, USA), nourseothricin (50 or 100 mg/L, GoldBio, USA), G418 (300 mg/L, Sigma-Aldrich), carbenicillin (300 mg/L, Teknova, USA), cefotaxime (300 mg/L, Sigma-Aldrich). All other chemicals were purchased through Sigma-Aldrich.

Induction medium (IM) was composed as follows: 1 g/L NH_4_Cl, 300 mg/L MgSO_4_·7H_2_O, 150 mg/L KCl, 10 mg/L CaCl_2_, 0.75 mg/L FeSO_4_·7H_2_O, 144 mg/L K_2_HPO_4_, 48 mg/L NaH_2_PO_4_, 3.9 g/L 2-(N-morpholino)ethanesulfonic acid (MES) (RPI, USA), 2 g/L D-glucose, 400 µM acetosyringone, 10 mg/L thiamine hydrochloride, pH adjusted to 5.5 with KOH. 2% Difco Bacto Agar (BD Biosciences) was used for induction medium plates. Medium was autoclaved before the addition of filter-sterilized stock solutions of thiamine (100 g/L in H_2_O) and acetosyringone (400 mM in DMSO (Sigma-Aldrich)). We employ a high acetosyringone concentration (400 µM) which we previously found to be effective in *Rhodotorula toruloides* IFO 0880 (NBRC 0880). Researchers should adjust this for their yeast of interest as it likely affects transformation efficiency in different hosts [15, 35].

### Strains and plasmids

All bacterial and yeast strains used in this work are listed in Table 1, and all plasmids are listed in Table 2. All plasmids generated in this paper were assembled using PCR and Gibson Assembly® and transformed into *E. coli* NEB-turbo using standard protocols [36]. Primers were purchased from Integrated DNA Technologies (IDT, USA). Plasmids were isolated using the QIAprep Spin Miniprep kit (Qiagen, USA). Plasmid sequences were verified using whole plasmid sequencing (Plasmidsaurus, USA). *A. tumefaciens* was routinely transformed via electroporation as described previously [37].

**Table 1:** Strains employed in this study.

Species	Source/Citation
*Agrobacterium tumefaciens* EHA105	[38]
*Escherichia coli* NEBturbo	NEB
*Rhodotorula toruloides* NBRC 0880	NBRC
*Saccharomyces cerevisiae* S288c	[39]
*Kluyveromyces marxianus* Km1	[40] ABF_015686
*Metschnikowia pulcherrima* 40–214 (ATCC 18406)	Phaff Collection, Davis, CA
*Botryozyma nematodophila* CBS 7442	CBS, [27]
*Komagataella phaffii* (*Pichia pastoris*) X-33	Invitrogen
*Ogataea parapolymorpha* DL-1 (ATCC 26012)	ATCC
*Lipomyces tetrasporus* NRRL Y-11562 (CBS 5910)	CBS, [41]

More details on providers of strains are available in Table S1 in S1 File.

**Table 2:** Plasmids used in this study.

Plasmid	JBEI Part ID	Source	NCBI
pVS1_R106H	JPUB_024140	[18]	
pVL_sR2rn_f14rg	JPUB_028034	This Work	PZ315364
pVR_sATn_fS3sg	JPUB_028038	This Work	PZ315359
pVR_sATon_fS3sg	JPUB_028036	This Work	PZ315361
pVR_sLRln_fLTsg	JPUB_028040	This Work	PZ315362
pVR_sATk_fS3sg	JPUB_028044	This Work	PZ315360
pVR_sATk_fS3sg_hom	JPUB_028042	This Work	PZ315363

All sequences are available through public-registry.jbei.org and NCBI

Colony PCR for *Botryozyma nematodophila* and *Metschnikowia pulcherrima* transformants were conducted using 2 × Phanta Flash Master Mix (Dye Plus) (Vazyme Biotech, CN). Colonies were picked with 20 µL pipettes and swirled in 20 µL of prepared PCR mix containing primers. PCR protocol and primer details are provided in Tables S3 and S4 in S1 File within the Supporting Information.

### Vacuum filtration *Agrobacterium-*mediated transformation

This describes a protocol for up to 10 total transformations. This can be scaled up using larger culture volumes and centrifugation formats.

**Day 1**: A single colony of *Agrobacterium* carrying a binary vector was inoculated into a culture tube containing 10 mL LB medium with kanamycin and cultured overnight (~16 hours, 30 °C, 200 RPM). The target yeast strain was inoculated from a single colony into a culture tube containing 10 mL YPD and grown in parallel.

**Day 2**: The overnight *Agrobacterium* culture was diluted to OD_600_ = 0.5 in 10 mL LB without antibiotics and grown at 30 °C with shaking until OD_600_ = 1.0 (~2.5 hours). Cells were harvested by centrifugation at 5,000 × g for 15 minutes in 15-mL conical tubes (Falcon, Corning, USA), washed once with 10 mL induction medium (IM), and resuspended in fresh IM to a final OD_600_ = 1.0. The culture was then incubated at 30 °C with shaking overnight for *vir*-gene induction. Serial dilutions of the overnight yeast culture were prepared in fresh YPD so that one dilution would reach OD_600_ = 1.0 the following day. The dilutions employed in this study ranged from starting OD_600_ values of 0.00001 to 0.1. Typically a starting OD_600_ between 0.00001 and 0.00005 yielded cultures of OD_600_ the following day (approx. 16 hours). For the slow growing strains, *L. tetrasporus* and *B. nematodophila*, a higher dilution series of OD_600_ 0.005–0.05 was employed.

**Day 3**: The OD_600_ of both the induced *Agrobacterium* and yeast cultures was measured. 1 mL of yeast culture (OD_600_ = 1.0) was transferred to a 1.5-mL microcentrifuge tube (Eppendorf, Germany) and pelleted at 5,000 × g for 5 minutes. The supernatant was aspirated, and the yeast pellet was resuspended in a volume of induced *Agrobacterium* culture yielding an equivalent OD_600_ ratio (e.g., 666 µL of OD 1.5 Agrobacterium culture). The combined cell suspension (1,000 µL) was applied in 250-µL increments onto a 0.45-µm mixed cellulose ester membrane filter (HAWP; MilliporeSigma, USA) under vacuum. The filter was transferred with sterile forceps, cell-side up, to an IM agar plate and incubated at 26 °C for 4 days.

**Day 7**: The filter was carefully removed from the IM plate with sterile forceps and placed into a 50-mL conical tube containing 5 mL of YPD. The tube was vortexed vigorously for 2 minutes to resuspend the cells. Serial dilutions were prepared, and aliquots were spread onto selective YPD agar plates supplemented with cefotaxime and carbenicillin to counter-select *Agrobacterium*. Plates were incubated at 30 °C for 2 days or until single colonies were visible.

Colonies were counted from the plates containing individual colonies with minimal overlap. Generally this was between 100 and 700 small colonies on a standard 90 mm diameter petri dish plate.

### Unfiltered spot-plating *Agrobacterium-*mediated transformation

Days 1 and 2 were performed identically to the vacuum filtration protocol described above.

**Day 3**: The OD_600_ of both the induced *Agrobacterium* and yeast cultures was measured. 1 mL of yeast culture (OD_600_ = 1.0) was transferred to a 1.5-mL microcentrifuge tube (Eppendorf, Hamburg, Germany) and pelleted at 5,000 × g for 5 minutes. The supernatant was aspirated, and the yeast pellet was resuspended in the induced *Agrobacterium* culture at an equivalent OD_600_ ratio (i.e., 1:1 by cell density). The combined suspension was then centrifuged at 10,000 × *g* for 5 minutes, the supernatant was aspirated, and the cells were resuspended in 20 µL of sterile deionized water. The concentrated cell mixture was spotted directly onto the surface of an IM agar plate and incubated at 26 °C for 4 days.

**Day 7**: The cell mixture was scraped from the surface of the IM agar using a 10 µL sterile loop (Thermofisher Scientific, USA) and resuspended in 1 mL YPD by swirling. Cell suspensions were vortexed until cell clumps were no longer visible, serial dilutions were prepared, and aliquots were spread onto selective YPD agar plates supplemented with cefotaxime and carbenicillin to counter-select *Agrobacterium*. Plates were incubated at 30 °C for 2 days or until single colonies were visible.

### Microscopy and spectrophotometry

For all cultures, OD_600_ was measured using a Spectramax M2 (Molecular Devices, USA). *Metschnikowia pulcherrima* and *Komagataella phaffii* (*Pichia pastoris*) were prone to forming flocs in liquid culture, confounding optical density measurements. For accurate measurements, these cultures were diluted into an EDTA solution (50 mM, pH = 8) and vortexed to disrupt flocs before reading.

For fluorescence measurements, 96 randomly selected colonies were used to inoculate a 96 deep-well plate, with each well containing 500 μL of YPD supplemented with 20 mg L^−1^ nourseothricin and 300 mg L^−1^ timentin. Cultures were grown overnight at 30 °C with shaking at 200 rpm. The next day, 150 μL of each culture was combined with 150 μL of fresh YPD in a 96-well plate, and both OD_600_ and GFP fluorescence were measured using a BioTek Synergy H1 microplate reader (excitation: 483 nm; emission: 512 nm). GFP fluorescence values were normalized by dividing the measured fluorescence by the corresponding OD600.

Colonies of yeast species were inoculated and cultured overnight in YPD (30 °C, 200 RPM). Cultures were spotted onto microscope slides (VWR) and examined under a VistaVision compound microscope (VWR, USA) with 10 x magnification eyepiece and 100 x magnification objective (1000 x total magnification). Images were taken with an iPhone 14 Plus (Apple, USA) main camera (12 MP, 26 mm, ƒ/1.5 aperture) held to the eyepiece lens without software magnification. Fluorescence microscopy was conducted with a Leica DM6B microscope.

## Supporting information

S1 Fig Fluorescence microscopy confirms successful transformation of Rhodotorula. Brightfield (left), fluorescence (middle), and brightfield/fluorescence overlay (right) images of a *Rhodotorula* transformant (top) compared to wildtype *Rhodotorula* (bottom). Fluorescence signal is observed only in the transformant, consistent with expression of the fluorescent marker.
(TIF)

S2 Fig Comparison of resuspension volume on the frequency of transformants (measured in CFU) for *Rhodotorula toruloides.* There was no statistically significant effect.
(TIF)

S3 Fig Colony PCR results for 8 random *Metschnikowia pulcherrima* and *Botryozyma nematodophila* transformants. A) *M*. *pulcherrima* transformed with pVR_sLRln_fS3sg. B) *B. nematodophila* transformed with pVR_sLRln_fS3sg. C) *B. nematodophila* transformed with pVR_sATon_fS3sg. D) *M*. *pulcherrima* transformed with pVR_sATon_fS3sg. E) *B. nematodophila* transformed with pVR_sATn_fS3sg. pVR_sATn_fS3sg and pVR_sATon_fS3sg transformants were subjected to PCR with primers rMRI132 and rMRI137 with a predicted amplicon of 661 bp and 666 bp respectively. pVR_sLRln_fS3sg transformants were subjected to PCR with primers rMRI207 and rMRI208 with a positive amplicon of 1,709 bp.
(TIF)

S4 Fig Microscopy of all yeasts investigated in this study. (TIF)

S1 File Supporting Tables. Table S1) Detailed list of yeasts investigated in this study. Table S2) Complete results from spot plating diverse ascomycetes. Table S3) Modified colony PCR protocol for *B. nematodophila* and *M. pulcherrima* with modifications from manufacturer’s instructions noted with asterisks. Table S4) Colony PCR primers used in this study, their templates, and amplicon lengths.
(XLSX)

## References

[1] Idnurm A, Bailey AM, Cairns TC, Elliott CE, Foster GD, Ianiri G, et al. A silver bullet in a golden age of functional genomics: the impact of Agrobacterium-mediated transformation of fungi. Fungal Biol Biotechnol. 2017;4:6. doi: 10.1186/s40694-017-0035-0 28955474 PMC5615635

[2] Zambryski P, Holsters M, Kruger K, Depicker A, Schell J, Van Montagu M, et al. Tumor DNA structure in plant cells transformed by A. tumefaciens. Science. 1980;209(4463):1385–91. doi: 10.1126/science.6251546 6251546

[3] Stachel SE, Messens E, Van Montagu M, Zambryski P. Identification of the signal molecules produced by wounded plant cells that activate T-DNA transfer in Agrobacterium tumefaciens. Nature. 1985;318(6047):624–9. doi: 10.1038/318624a0

[4] Gelvin SB. Traversing the Cell: Agrobacterium T-DNA’s Journey to the Host Genome. Front Plant Sci. 2012;3:52. doi: 10.3389/fpls.2012.00052 22645590 PMC3355731

[5] de Framond AJ, Barton KA, Chilton MD. Mini–ti: A new vector strategy for plant genetic engineering. Nat Biotechnol. 1983;1(3):262–9.

[6] Hoekema A, Hirsch PR, Hooykaas PJJ, Schilperoort RA. A binary plant vector strategy based on separation of vir- and T-region of the Agrobacterium tumefaciens Ti-plasmid. Nature. 1983;303(5913):179–80. doi: 10.1038/303179a0

[7] Bevan M. Binary Agrobacterium vectors for plant transformation. Nucleic Acids Res. 1984;12(22):8711–21. doi: 10.1093/nar/12.22.8711 6095209 PMC320409

[8] Gelvin SB. Agrobacterium-mediated plant transformation: the biology behind the “gene-jockeying” tool. Microbiol Mol Biol Rev. 2003;67(1):16–37, table of contents. doi: 10.1128/MMBR.67.1.16-37.2003 12626681 PMC150518

[9] Bundock P, den Dulk-Ras A, Beijersbergen A, Hooykaas PJ. Trans-kingdom T-DNA transfer from Agrobacterium tumefaciens to Saccharomyces cerevisiae. EMBO J. 1995;14(13):3206–14. 10.1002/j.1460-2075.1995.tb07323.x PMC394382 7621833

[10] Smith MD, Guild WR. Improved method for conjugative transfer by filter mating of Streptococcus pneumoniae. J Bacteriol. 1980;144(1):457–9. doi: 10.1128/jb.144.1.457-459.1980 6998966 PMC294685

[11] Piers KL, Heath JD, Liang X, Stephens KM, Nester EW. Agrobacterium tumefaciens-mediated transformation of yeast. Proc Natl Acad Sci USA. 1996;93(4):1613–8. 10.1073/pnas.93.4.1613 PMC39990 8643679

[12] de Groot MJ, Bundock P, Hooykaas PJ, Beijersbergen AG. Agrobacterium tumefaciens-mediated transformation of filamentous fungi. Nat Biotechnol. 1998;16(9):839–42. doi: 10.1038/nbt0998-839 9743116

[13] Michielse CB, Hooykaas PJJ, van den Hondel CAMJJ, Ram AFJ. Agrobacterium-mediated transformation as a tool for functional genomics in fungi. Curr Genet. 2005;48(1):1–17. doi: 10.1007/s00294-005-0578-0 15889258

[14] Chong NF, Idnurm A, Nugent BC. Genetic Transformation of Cryptococcus Species with Agrobacterium Transfer DNA. Methods Mol Biol. 2024;2775:81–90. doi: 10.1007/978-1-0716-3722-7_6 38758312

[15] Liu Y, Koh CMJ, Yap SA, Du M, Hlaing MM, Ji L. Identification of novel genes in the carotenogenic and oleaginous yeast Rhodotorula toruloides through genome-wide insertional mutagenesis. BMC Microbiol. 2018;18(1):14. doi: 10.1186/s12866-018-1151-6 29466942 PMC5822628

[16] Nora LC, Wehrs M, Kim J, Cheng J-F, Tarver A, Simmons BA, et al. A toolset of constitutive promoters for metabolic engineering of Rhodosporidium toruloides. Microb Cell Fact. 2019;18(1):117. doi: 10.1186/s12934-019-1167-0 31255171 PMC6599526

[17] Coradetti ST, Pinel D, Geiselman GM, Ito M, Mondo SJ, Reilly MC. Functional genomics of lipid metabolism in the oleaginous yeast Rhodosporidium toruloides. eLife. 2018;7. doi: 10.7554/eLife.32101 PMC5922974 29521624

[18] Szarzanowicz MJ, Busche M, Dai Z, Lapp N, Geiselman GM, Jia J. Binary vector origin predictably determines Agrobacterium-mediated transformation outcome across eukaryotic kingdoms. BioRxiv. 2025.

[19] Bundock P, Mróczek K, Winkler AA, Steensma HY, Hooykaas PJ. T-DNA from Agrobacterium tumefaciens as an efficient tool for gene targeting in Kluyveromyces lactis. Mol Gen Genet. 1999;261(1):115–21. doi: 10.1007/s004380050948 10071217

[20] Hoshida H, Murakami N, Suzuki A, Tamura R, Asakawa J, Abdel-Banat BMA, et al. Non-homologous end joining-mediated functional marker selection for DNA cloning in the yeast Kluyveromyces marxianus. Yeast. 2014;31(1):29–46. doi: 10.1002/yea.2993 24307396

[21] Lu M, Billerbeck S. Improving homology-directed repair by small molecule agents for genetic engineering in unconventional yeast?-Learning from the engineering of mammalian systems. Microb Biotechnol. 2024;17(2):e14398. doi: 10.1111/1751-7915.14398 38376092 PMC10878012

[22] Dai Z, Deng S, Culley DE, Bruno KS, Magnuson JK. Agrobacterium tumefaciens-mediated transformation of oleaginous yeast Lipomyces species. Appl Microbiol Biotechnol. 2017;101(15):6099–110. doi: 10.1007/s00253-017-8357-7 28631219

[23] Gordon ZB, Soltysiak MPM, Leichthammer C, Therrien JA, Meaney RS, Lauzon C. Development of a transformation method for Metschnikowia borealis and other CUG-serine yeasts. Genes. 2019;10(2). 10.3390/genes10020078 PMC6409616 30678093

[24] Wu S, Letchworth GJ. High efficiency transformation by electroporation of Pichia pastoris pretreated with lithium acetate and dithiothreitol. Biotechniques. 2004;36(1):152–4. doi: 10.2144/04361DD02 14740498

[25] Faber KN, Haima P, Harder W, Veenhuis M, AB G. Highly-efficient electrotransformation of the yeast Hansenula polymorpha. Curr Genet. 1994;25(4):305–10. doi: 10.1007/BF00351482 8082173

[26] PhyloT: a phylogenetic tree generator. https://phylot.biobyte.de/ Accessed 2026 April 2.

[27] Smith MT, Shann C, Batenburg-van der Vegte WH, Schmitt R, Wehrli E, Roeijmans HJ. Botryozyma nematodophila gen. nov., spec. nov. (Candidaceae). Antonie Van Leeuwenhoek. 1992;61(4):277–84. 10.1007/BF00713936 1497332

[28] Larini I, Ferrara M, Troiano E, Gatto V, Mulè G, Vitulo N, et al. Unlocking the potential of Metschnikowia pulcherrima: a dive into the genomic and safety characterization of four plant-associated strains. Appl Microbiol Biotechnol. 2025;109(1):128. doi: 10.1007/s00253-025-13515-0 40439729 PMC12122572

[29] Liu J, Rygała A, Zhang B, Kręgiel D. Potential of Metschnikowia yeasts in green applications: a review. Front Microbiol. 2025;16:1652494. 10.3389/fmicb.2025.1652494 PMC12528079 41113634

[30] Prillinger H, Oberwinkler F, Umile C, Tlachac K, Bauer R, Dörfler C, et al. Analysis of cell wall carbohydrates (neutral sugars) from ascomycetous and basidiomycetous yeasts with and without derivatization. J Gen Appl Microbiol. 1993;39(1):1–34.

[31] Buck JW, Andrews JH. Attachment of the yeast Rhodosporidium toruloides is mediated by adhesives localized at sites of bud cell development. Appl Environ Microbiol. 1999;65(2):465–71. doi: 10.1128/AEM.65.2.465-471.1999 9925569 PMC91048

[32] Dashko S, Zhou N, Compagno C, Piškur J. Why, when, and how did yeast evolve alcoholic fermentation?. FEMS Yeast Research. 2014;14(6):826–32. 10.1111/1567-1364.12161 PMC4262006 24824836

[33] Karim A, Gerliani N, Aïder M. Kluyveromyces marxianus: An emerging yeast cell factory for applications in food and biotechnology. Int J Food Microbiol. 2020;333:108818. 10.1016/j.ijfoodmicro.2020.108818 32805574

[34] Sipiczki M. Metschnikowia pulcherrima and related pulcherrimin-producing yeasts: fuzzy species boundaries and complex antimicrobial antagonism. Microorganisms. 2020;8(7). 10.3390/microorganisms8071029 PMC7409158 32664630

[35] Fu J, Brockman NE, Wickes BL. Optimizing transformation frequency of Cryptococcus neoformans and Cryptococcus gattii using Agrobacterium tumefaciens. J Fungi (Basel). 2021;7(7). 10.3390/jof7070520 PMC8305055 34209781

[36] Gibson DG, Young L, Chuang R-Y, Venter JC, Hutchison CA 3rd, Smith HO. Enzymatic assembly of DNA molecules up to several hundred kilobases. Nat Methods. 2009;6(5):343–5. doi: 10.1038/nmeth.1318 19363495

[37] Kámán-Tóth E, Pogány M, Dankó T, Szatmári Á, Bozsó Z. A simplified and efficient Agrobacterium tumefaciens electroporation method. 3 Biotech. 2018;8(3):148. doi: 10.1007/s13205-018-1171-9 29487777 PMC5821610

[38] Hood EE, Gelvin SB, Melchers LS, Hoekema A. NewAgrobacterium helper plasmids for gene transfer to plants. Transgenic Research. 1993;2(4):208–18. doi: 10.1007/bf01977351

[39] Mortimer RK, Johnston JR. Genealogy of principal strains of the yeast genetic stock center. Genetics. 1986;113(1):35–43. doi: 10.1093/genetics/113.1.35 3519363 PMC1202798

[40] Cernak P, Estrela R, Poddar S, Skerker JM, Cheng Y-F, Carlson AK. Engineering Kluyveromyces marxianus as a robust synthetic biology platform host. MBio. 2018;9(5). 10.1128/mBio.01410-18 PMC6156195 30254120

[41] Nieuwdorp PJ, Bos P, Slooff WC. Classification of Lipomyces. Antonie Van Leeuwenhoek. 1974;40(2):241–54. 10.1007/BF00394382 4599856

